# Is Orthorexia Nervosa a Non-specific Eating Disorder or a Disease in the Spectrum of Obsessive-Compulsive Disorder?

**DOI:** 10.7759/cureus.38451

**Published:** 2023-05-02

**Authors:** Nazan Dolapoglu, Duygu Ozcan, Rıza Gokcer Tulaci

**Affiliations:** 1 Department of Psychiatry, Balikesir University Faculty of Medicine, Balıkesir, TUR; 2 Department of Psychiatry, Balikesir University Faculty of Medicine, Balikesir, TUR

**Keywords:** conscious awareness scale, body perception scale, health anxiety, eating disorders, orthorexia nervosa

## Abstract

Background: In recent years, there has been a dramatic increase in awareness in society that healthy nutrition has positive effects on health. However, obsession with these behaviors towards healthy foods causes negative effects on health and quality of life.

Aim: The aim of this study was to elucidate the relationship between the incidence of orthorexia nervosa (ON) in medical school students and the level of conscious awareness, obsessive-compulsive disorder, eating attitudes and behaviors, health anxiety, and body image to clarify the unclear issues in the literature, such as whether orthorexia nervosa is among the psychological disorders, "where" it will take place, and which diagnoses it can be associated with.

Methods: Students between the 1^st^ and 6^th^ grades of medical school were invited to participate in this research. The Sociodemographic Data Form, Maudsley Obsessive-Compulsive Question Index (MOCI), Eating Attitude Test Short Form (EAT SF-26), Health Anxiety Inventory-Weekly Short Form (HAI-SF), ORTO-11 scale, Body Perception Scale, and Conscious Awareness Scale have been applied to the students.

Results: In univariate analysis, the eating disorder scale, body image scale, and awareness scale total scores all had an impact on orthorexia. Each increase in the eating disorder scale score increased the diagnosis of orthorexia 1.07 times, while each increase in the body image scale score increased the diagnosis of orthorexia 1.09 times. Additionally, each increase in the conscious awareness scale score decreased the diagnosis of orthorexia by 0.92 times. When all variables were re-evaluated in the multivariate analysis, it was seen that the total scores of the body image scale and conscious awareness scale affected the diagnosis of orthorexia. There was a weak inverse relationship between the orthorexia scale score and only the health anxiety inventory total score (p<0.05).

Conclusion: Regarding the outcomes of this research, one can say that orthorexia affected the eating disorder scale, body image scale, and awareness scale total scores. While the increase in the eating disorder and body image scale scores increased orthorexia, the increase in the conscious awareness scale score had a decreasing effect.

## Introduction

In recent years, there has been a dramatic increase in awareness in society that healthy nutrition has positive effects on health. However, obsession with these behaviors towards healthy foods causes negative results in health and quality of life. This condition is called "orthorexia nervosa" (ON). ON is a new concept among disease groups called eating disorders [1]. Unlike anorexia and bulimia, people with orthorexia may experience feelings of guilt and a lack of motivation about not eating healthy. As the obsession with healthy food becomes excessive, psychological and social complications arise as a result [2].

The obsessive and compulsive features of orthorexia nervosa become pathological over time and dominate one's life. The effort to consume healthy and high-quality foods is the main cause of this disorder. Concentration on biologically pure foods and the shops that sell them leads to a pathological obsession and a special lifestyle. Efforts to comply with dietary rules lead to intense anxiety and feelings of guilt and shame, which continue with more stringent dietary restrictions [3]. It is thought that there is a similarity between these attitudes and obsessive-compulsive behaviors. However, the most important point where orthorexia differs from obsessive-compulsive disorder is that the content of obsessions in orthorexia is egosyntonic [4]. In other words, the orthorexic person finds his own attitudes, tendencies, behaviors, and thoughts acceptable and accepts them as a normal reflection of his personality. Also, high levels of positive correlations were found between eating disorders and ON symptoms [5]. On the other hand, concerns about weight gain in ON are thought to diverge from those in AN and BN. People with AN and BN eating disorders focus on their quantity. However, orthorexic people are obsessed with the quality of the food they eat [6]. It is still unclear whether ON is a type of eating disorder or a dimension of an eating pattern.

Cognitions and concerns about healthy eating, the right choice of food, or situations where proper nutrition has become the most important part of their lives may lead to this attitude becoming pathological. People are fed only certain foods, and this sometimes causes a deficiency of important nutrients. In addition, this may change eating behavior by causing significant dietary restrictions, or ultimately, many things become important about what people eat, how to eat, and how to prepare, thus affecting people in many areas (for example, being socially separated from family members or friends who do not share similar views about food).

The concept of orthorexia nervosa was first coined in 1997 by Steven Bratman. Bratman used the term "orthorexia nervosa" to describe the pathology associated with healthy food consumption, as it means "ortho" or "true" [7]. Bratman describes the concept of orthorexia in his book "Health Food Junkies" and defines diets as a disease that people do to feel more attentive and clean. In the following years, orthorexia nervosa entered the English language and affected the whole world [8]. Clinicians and scientists still carry on the debate on whether orthorexia is a real and unique disorder and whether it is worth its own categorization in the "Diagnostic and Statistical Manual of Mental Disorders (DSM-5)" together with eating disorders (anorexia nervosa, bulimia nervosa, and eating disorders not otherwise specified). On the one hand, eating disorder experts in the United Kingdom argue that orthorexia is not currently identified with an eating disorder because it does not begin with low self-esteem, but it may in time result in an eating disorder as the diet becomes more refined and compulsive [9].

Although the exact figures are not known, there are different results regarding the prevalence. In a study conducted in the United States, the prevalence of orthorexia nervosa was found to be 6.9-57.6% in the general population, thus reaching up to 81.1% in special groups. In recent years, the incidence of orthorexia has increased. The reason for this is the perception of beauty associated with thinness, the media's interest in diet and the news about the ingredients of the products, and the fact that some products contain additives, dyes, and carcinogenic substances. The higher-risk groups for orthorexia nervosa are women, adolescents, people who practice sports (bodybuilding, athletics), medical physicians and medical students, dieticians, as well as performance artists [10].

Clinical observations, theoretical views, and research findings suggest that health anxiety, body image, and obsessive beliefs may be associated with orthorexia nervosa and disordered eating attitudes. However, when these factors are taken together, it is not known which ones are important in explaining different eating patterns. In this research, we aimed to elucidate the relationship between the incidence of orthorexia nervosa in medical school students and the level of conscious awareness, obsessive-compulsive disorder, eating attitudes and behaviors, health anxiety, and body image to clarify the unclear issues in the literature, such as whether orthorexia nervosa is among the psychological disorders, "where" it will take place, and which diagnoses it can be associated with. The hypothesis of our study is to clarify the unclear issues in the literature, such as whether ON is among the psychological disorders, "where" it will take place, and which diagnoses it can be associated with.

## Materials and methods

This is a cross-sectional and retrospective study. Two hundred students between the 1st and 6th grades of the Balikesir University Medical Faculty have been invited to participate in this research. After all medical faculty students were informed about the study, those who volunteered to participate were included in it. All procedures followed were in accordance with the ethical standards of the responsible committee on human experimentation (institutional and national) and with the Helsinki Declaration of 1975, as revised in 2008. Informed consent was obtained from all participants. The study has been approved by the ethics committee.

The sociodemographic data forms, Maudsley Obsessive-Compulsive Question Index (MOCI), Eating Attitude Test Short Form (EAT SF-26), Health Anxiety Inventory-Weekly Short Form (HAI-SF), Body Perception Scale, Conscious Awareness Scale, and ORTO-11 Scale, have been applied to the students. The ORTO-15 is a 15-item self-administered questionnaire used in the diagnosis of ON. The responses designated as differential criteria for orthorexia are graded by 1 point, while answers indicating a normal eating-behavior tendency earn 4 points. The lowest possible score on the scale is 15 points, and the highest is 60 points. An overall scale score below 40 points is considered indicative of orthorexia, while higher scores indicate normal eating behavior [6]. The reliability and validity study of the Turkish version of the ORTO-15 was performed by Arusoglu. It was adapted into Turkish as ORTO-11 for evaluation in Turkey.

As a result of the evaluation, the demographic data of students with and without high orthorexia nervosa tendency have been compared, eating attitudes and behaviors, OCD symptoms, health anxiety level, conscious awareness level, and body perception of students with high orthorexia nervosa tendency have been analyzed, and the predictive power of the meaningful values has been interpreted. Students under the age of 18, individuals with psychiatric disorders, and primary or acquired neurological diseases that may affect cognitive abilities (stroke, dementia, head trauma, cranial operations) were excluded from the study. This study lasted two months, from March 1, 2022 to April 30, 2022.

Statistical analysis

Patient data collected within the scope of the study were analyzed with the IBM Statistical Package for the Social Sciences (SPSS) for Windows 23.0 (IBM Corp., Armonk, NY) package program. Frequency and percentage were given for categorical data and median, minimum, and maximum descriptive values for continuous data. "Mann Whitney U-Test" was utilized for comparisons between groups, and "Chi-square or Fisher's Exact Test" was used for the comparison of categorical variables. "Logistic regression analysis" was used to determine the risk factors affecting the diagnosis of orthorexia, and "Spearman correlation analysis" was used to evaluate the relationship between scale scores. The results were considered statistically significant when the p-value was less than 0.05.

## Results

Within the scope of the study, a total of 142 individuals, 76 (53.5%) with orthorexia and 66 (46.5%) in the control group, were included in the study. While 52.8% (n=75) of the participants were female, 47.2% (n=67) were male. The ages of the participants ranged from 18 to 31 years old, with an average age of 22 years old. The distribution of demographic characteristics according to the status of being diagnosed with orthorexia among the participants is denoted in Table 1. When the table was examined, a statistically significant difference was found between the two groups in terms of age, the class they studied, and with whom they lived (p<0.05).

**Table 1:** Distribution of participants' scale and scale subgroup scores

	Total (N=142)	Orthorexia (n=76)	Control (n=66)	p-value
	n (%) or median (min-max)	n (%) or median (min-max)	n (%) or median (min-max)	
Obsessive-compulsive disorder total	10 (0–31)	10.5 (1–29)	10 (0–31)	0.294
Control	2 (0–9)	2 (0–8)	2 (0–9)	0.327
Cleaning	3 (0–10)	3 (0–9)	3 (0–10)	0.569
Slowness	1 (0–6)	1 (0–6)	1 (0–5)	0.557
Doubt	2 (0–7)	3 (0–7)	2 (0–7)	0.301
Rumination	2 (0–9)	2.5 (0–9)	2 (0–9)	0.299
Eating disorder total	7 (0–39)	10 (0–39)	6 (0–23)	0.013
Dieting	2 (0–21)	4 (0–21)	1.5 (0–17)	0.007
Bulimia and eating obsession	1.5 (0–12)	2 (0–10)	1 (0–12)	0.032
Oral control behavior	3 (0–16)	2 (0–16)	3 (0–12)	0.661
Health anxiety inventory total	15 (3–42)	15.5 (4–36)	15 (3–42)	0.899
Hypersensitivity to bodily symptoms and anxiety	12 (2–32)	12 (4–27)	12 (2–32)	0.883
Negative consequences of the disease	3 (0–10)	3 (0–10)	3.5 (0–10)	0.372
Orthorexia total	25.5 (15–40)	23 (15–27)	30 (28–40)	<0.001
Cognitive-rational domain	10 (5–16)	9 (5–12)	12 (8–16)	<0.001
Orthorexia clinical field	9 (5–12)	8 (5–10)	10 (8–12)	<0.001
Orthorexia emotional area	7 (3–12)	5.5 (3–9)	9 (6–12)	<0.001
Body Perception Scale total	75 (5–150)	87.5 (40–150)	60 (5–88)	<0.001
Conscious Awareness Scale total	68 (9–174)	56.5 (29–87)	89.5 (9–174)	<0.001

The distribution of total and sub-dimension scores of the scales according to the diagnosis groups of the participants is elaborated in Table 2. When the table was examined, it was observed that there was a statistically significant difference between the two groups in the total score of eating disorders and the sub-dimensions of dieting, bulimia, and eating obsession (p<0.05). It is noteworthy that the scores of the orthorexia group were higher than the scores of the control group. Similarly, there was a statistically significant difference between the two groups in the body image scale and awareness scale scores, while the body image scale score in the orthorexia group and the awareness scale score in the control group were found to be higher.

**Table 2:** Evaluation of risk factors affecting orthorexia

	Univariant		Multivariant	
	Odds ratio (95% CI)	p-value	Odds ratio (95% CI)	p-value
Obsessive-compulsive disorder	1.02 (0.97–1.08)	0.379	1.01 (0.92–1.12)	0.808
Eating disorder	1.07 (1.02–1.13)	0.010	1.08 (0.99–1.18)	0.097
Health anxiety inventory	0.99 (0.94–1.05)	0.819	1.00 (0.90–1.11)	0.954
Body Perception Scale	1.09 (1.06–1.12)	<0.001	1.07 (1.04–1.11)	<0.001
Conscious Awareness Scale	0.92 (0.90–0.95)	<0.001	0.94 (0.91–0.97)	<0.001

The distribution of risk factors affecting the diagnosis of orthorexia among the participants in the study is shown in Table 3. When the table was examined, it was determined that orthorexia was affected by the eating disorder scale, body image scale, and awareness scale total scores in univariate analysis. While the increase in the eating disorder and body image scale scores increased orthorexia, it was seen that the increase in the conscious awareness scale score had a decreasing effect. It was determined that each increase in the eating disorder scale score increased the diagnosis of orthorexia 1.07 times, while each increase in the body image scale score increased the diagnosis of orthorexia 1.09 times. It was observed that each increase in the conscious awareness scale score decreased the diagnosis of orthorexia by 0.92 times. When all variables were re-evaluated in the multivariate analysis, it was seen that the total scores of the body image scale and conscious awareness scale affected the diagnosis of orthorexia.

**Table 3:** Evaluation of the relationship between scale scores of the orthorexia group

		Orthorexia Total Score
Obsessive-compulsive disorder total	Correlation coefficient	−0.122
	p-value	0.292
	N	76
Eating disorder total	Correlation coefficient	−0.169
	p-value	0.144
	N	76
Health anxiety inventory total	Correlation coefficient	−0.242
	p-value	0.035
	N	76
Body Perception Scale total	Correlation coefficient	−0.052
	p-value	0.653
	N	76
Conscious Awareness Scale total	Correlation coefficient	0.152
	p-value	0.190
	N	76

The distribution of the relationship between orthorexia total scores and other scale total scores for individuals diagnosed with orthorexia is given in Table 4. When the table was examined, it was seen that there was a weak inverse relationship between the orthorexia scale score and only the health anxiety inventory total score (p<0.05).

**Table 4:** Distribution of participants' demographic findings

	Total (N=142)	Orthorexia (n=76)	Control (n=66)	p-value
	n (%) or Median (Min-Max)	n (%) or Median (Min-Max)	n (%) or Median (Min-Max)	
Gender				0.101
Woman	75 (52.8)	45 (59.2)	30 (45.5)	
Male	67 (47.2)	31 (40.8)	36 (54.5)	
Age, year	22 (18–31)	21 (18–31)	23 (20–27)	<0.001
Class				<0.001
1^st^ grade	22 (15.5)	22 (28.9)	0 (0)	
2^nd^ grade	10 (7)	10 (13.2)	0 (0)	
3^rd^ grade	45 (31.7)	12 (15.8)	33 (50)	
4^th^ grade	9 (6.3)	9 (11.8)	0 (0)	
5^th^ grade	52 (36.6)	19 (25)	33 (50)	
6^th^ grade	4 (2.8)	4 (5.3)	0 (0)	
Height, cm	170 (153–190)	170 (153–188)	171 (155–190)	0.450
Kilo, kg	65 (40–145)	62.5 (49–105)	65 (40–145)	0.443
BMI, kg/cm^2^	22.4 (16.2–42.8)	22.4 (17.5–33.2)	22.1 (16.2–42.8)	0.867
The weight you want to be	60 (43–100)	60 (48–90)	67 (43–100)	0.351
Place of residence				0.151
Bay	4 (2.8)	4 (5.3)	0 (0)	
District	21 (14.8)	10 (13.2)	11 (16.7)	
Province	117 (82.4)	62 (81.6)	55 (83.3)	
Lives with				0.002
With my family	32 (22.5)	17 (22.4)	15 (22.7)	
With my roommate	18 (12.7)	9 (11.8)	9 (13.6)	
Home alone	56 (39.4)	21 (27.6)	35 (53)	
In the state dormitory	15 (10.6)	12 (15.8)	3 (4.5)	
In private dormitory	21 (14.8)	17 (22.4)	4 (6.1)	
Mental disorders in the family	22 (15.5)	13 (17.1)	9 (13.6)	0.736
Smoking	21 (14.8)	7 (9.2)	14 (21.2)	0.076
Alcohol use	36 (25.5)	17 (22.7)	19 (28.8)	0.523
Mother education level				0.628
Illiterate	4 (2.8)	3 (3.9)	1 (1.5)	
Literate‎/primary school	46 (32.4)	27 (35.5)	19 (28.8)	
Middle school-high school	49 (34.5)	24 (31.6)	25 (37.9)	
College	43 (30.3)	22 (28.9)	21 (31.8)	
Father's education level				0.710
Illiterate	1 (0.7)	1 (1.3)	0 (0)	
Literate‎/primary school	24 (16.9)	13 (17.1)	11 (16.7)	
Middle school-high school	45 (31.7)	22 (28.9)	23 (34.8)	
College	72 (50.7)	40 (52.6)	32 (48.5)	
Any disease diagnosed by a doctor	24 (16.9)	16 (21.1)	8 (12.1)	0.233
Regular drug therapy	23 (16.2)	15 (19.7)	8 (12.1)	0.317
Regular dietary therapy	5 (3.5)	4 (5.3)	1 (1.5)	0.372
Vitamin support				0.306
Yes	17 (12)	12 (15.8)	5 (7.6)	
Sometimes	51 (35.9)	27 (35.5)	24 (36.4)	
Regular sport	55 (38.7)	33 (43.4)	22 (33.3)	0.218

The correlation coefficient could be elaborated as being weak about being between 0.00 and 0.29, low about being between 0.30 and 0.49, medium about being between 0.50 and 0.69, strong about being between 0.70 and 0.89, and very strong about being between 0.90 and 1.00.

## Discussion

According to the results of our study, orthorexia was affected by the total scores of the eating disorder scale, body image scale, and awareness scale in univariate analysis. Each increase in the eating disorder scale score increased the diagnosis of orthorexia 1.07 times, while each increase in the body image scale score increased the diagnosis of orthorexia 1.09 times. Additionally, each increase in the conscious awareness scale score decreased the diagnosis of orthorexia by 0.92 times. When all variables were re-evaluated in the multivariate analysis, it was seen that the total scores of the body image scale and conscious awareness scale affected the diagnosis of orthorexia. There was a weak inverse relationship between the orthorexia scale score and only the health anxiety inventory total score (p<0.05).

A healthy lifestyle and diet, which are one of the foundations of health attributes, can become an unhealthy, life-threatening situation after a while. ON negatively affects the physical health of the person as well as their interpersonal relationships, stress management, and mental health. The etiology, epidemiology, and treatment approaches of this disorder are not fully known because studies on ON are not yet sufficient.

Zamora et al. stated that the obsessive-compulsive mechanisms and personality traits of patients with ON were similar to those of patients with the restrictive anorexia nervosa type [11]. Arusoğlu et al. found that deterioration in eating attitudes and obsessive-compulsive symptoms were associated with orthorexic tendencies [12]. Women, adolescents, individuals doing sports, medical students, healthcare professionals, and dietitians are considered to be the main risk groups for orthorexia nervosa [13]. Some researchers suggest that health anxiety is related to orthorexia [14].

Considering the relationship between orthorexia nervosa and body image, it was found that preoccupations with body appearance and anxiety about being overweight triggered orthorexia nervosa. It has been suggested that excessive focus on appearance and fear of being overweight may be the hidden motivation behind healthy diet preoccupation [15].

It is controversial whether orthorexia should be interpreted as a separate disorder or as a subset of obsessive-compulsive disorder or anorexia nervosa. Among the common features of orthorexia and anorexia nervosa, significant weight loss, increased anxiety, perfectionism, and an effort to keep control can be counted. While anorexia nervosa and bulimia nervosa elaborate eating disorders in a quantitative context (e.g., the amount of food consumed), orthorexia nervosa is seen in a qualitative context (the quality of food consumed) [2]. In our study, each increase in the eating disorder scale score increased the diagnosis of orthorexia 1.07 times, while each increase in the body image scale score increased the diagnosis of orthorexia 1.09 times. It was observed that each increase in the conscious awareness scale score decreased the diagnosis of orthorexia by 0.92 times.

In the association of orthorexia nervosa with obsessive-compulsive disorder, some obsessive tendencies are also observed in individuals. In addition, these individuals show intense anxiety about contamination, ritualized eating and arranging food, and recurrent intrusive thoughts about food and health. The most important difference between orthorexia and obsessive-compulsive disorder is that the content of the obsessions in orthorexia is not alien to the ego but compatible with the ego. Some diagnostic criteria have also been developed for ON previously [16,17]. In our study, we found a significant difference between the two groups in the total score of eating disorders and the sub-dimensions of dieting and bulimic behaviors like frequent binges, fear of losing control, extreme weight-control measures, and overt concern about body weight.

Inconsistent results emerge when investigating sex differences in orthorexia nervosa. This may be due to the differences in sampling and data collection tools. In addition, Oberle et al. mention the difficulty of detecting gender differences due to the lack of experience working with clinically diagnosed individuals, as ON is not yet included in the DSM-5 [18].

In some studies conducted in our country and around the world, it has been found that women may have more orthorexic tendencies than men. Donini et al. stated that the orthorexic tendency was higher in males. In addition to the different findings in the literature about ON, there are contradicting results in some studies on whether there is a significant relationship between gender and ON [6].

Barnes and Caltabiano found that high scores obtained from the body image scale were predictors of ON [15]. In addition, Barthels et al. emphasized that individuals prone to orthorexia have very rigid thoughts not only about healthy eating but also about a healthy body image [19]. Similarly, in our study, there was a statistically significant difference between the two groups in the body image scale and awareness scale scores, while the body image scale score in the orthorexia group and the awareness scale score in the control group were found to be higher.

A positive relationship was also found between orthorexia and health anxiety. This means that as orthorexic tendencies increase, health-related anxiety and worry also increase. It can be concluded that as the concern about healthy, pure food and consuming it increases, the concerns about health also increase. Toth-Kiraly et al. reached a similar conclusion in their study. According to this study, the more people worry about their health and bodily functions, the more they focus on a strict diet and strict physical activity [20]. To the best of our knowledge, this is the only study that evaluates many parameters together, such as eating attitudes, body perception, obsessive-compulsive symptoms, health anxiety, and consciousness awareness.

## Conclusions

Orthorexia nervosa involves obsessive thoughts about healthy eating and distress related to this obsession. There is still dispute over whether ON should be considered on the obsessive-compulsive spectrum, the eating disorder spectrum, or as its own disorder. Regarding the outcomes of this research, one can say that the orthorexia-affected eating disorder scale, body image scale, and awareness scale total scores. While the increase in the eating disorder and body image scale scores increased orthorexia, it was seen that the increase in the conscious awareness scale score had a decreasing effect.
